# Is the incidence rate of colorectal cancer increasing in Mozambique?

**DOI:** 10.3332/ecancer.2024.1693

**Published:** 2024-04-11

**Authors:** Carlos Selemane, Josefo Ferro, Cesaltina Lorenzoni, Carla Carrilho, Mamudo Rafik Ismail, Max Parkin, Lúcio Lara Santos

**Affiliations:** 1 Department of Surgery, Maputo Central Hospital, Av Agostinho Neto n° 164, Maputo 1164, Mozambique; 2 Department of Pathology, Beira Central Hospital, Av Mártires da Revolução nº 727, Beira, Mozambique; 3 Department of Pathology, Faculty of Medicine, Eduardo Mondlane University, and Maputo Central Hospital, Av Agostinho Neto n° 164, Maputo 1164, Mozambique; 4 African Cancer Registry Network, Prama House, 267 Banbury Road, Oxford OX2 7HT, UK; 5 Experimental Pathology and Therapeutics Research Group and Surgical Oncology Department, Portuguese Institute of Oncology, Dr António Bernardino de Almeida Street, Porto 4200-072, Portugal; 6 School of Medicine and Biomedical Sciences, Fernando Pessoa University, Av Fernando Pessoa 150, S. Gondomar 4420-096, Portugal

**Keywords:** colorectal cancer, Mozambique, incidence rate, developing countries

## Abstract

**Background:**

Colorectal cancer (CRC) is a significant global health concern, ranking as the third most common cancer and the second leading cause of cancer-related deaths. However, in Africa, CRC is the fifth most common invasive malignancy. Limited data hinder our understanding of the evolving burden of CRC in sub-Saharan Africa. This study explores CRC trends in Mozambique, utilising data from population-based oncological registries.

**Methods:**

CRC data were gathered from Beira and Maputo population-based cancer registries, along with supplementary information from pathology-based and hospital-based registries. Comparative analyses were performed across different time periods, focusing on trends and epidemiological characteristics.

**Results:**

Incidence rates of CRC in Maputo and Beira were relatively low historically. However, data from recent years showed an increase, especially in age groups above 50. Analyses from pathology-based and hospital-based registries affirmed the rising trend. The age-standardised incidence rate in Maputo (2015–2017) was 3.17 for males and 2.55 for females. Beira exhibited increasing rates between 2009 and 2020, particularly in individuals aged 50 and above.

**Conclusion:**

The study reveals an emerging burden of CRC in Mozambique, challenging the perception of low incidence. The rising trend underscores the necessity for tailored interventions, emphasizing early diagnosis, preventive strategies, and investments in healthcare infrastructure to address the increasing CRC burden in the region.

## Background

Colorectal cancer (CRC) is globally ranked as the third most common cancer and the second leading cause of cancer-related deaths [1]. However, in Africa, CRC ranks as the fifth most common invasive malignancy [2]. In Sub-Saharan Africa, the cumulative risk (for ages 0–74) of the disease varies across cancer registries, ranging from less than 0.2% in Gulu, Uganda, to over 3.6% in Reunion, France, for men; and from less than 0.2% in Gulu, Uganda, to 3.5% in Reunion, France, and 3.6% in Seychelles for women [3].

Numerous studies have documented increased CRC incidence rates in selected sub-Saharan African countries, including Uganda and South Africa [4–6]. Chokunonga *et al* [7] demonstrated that among the black population in Zimbabwe, age-standardised incidence rates per 100,000 men and women increased by approximately 4% annually during 1991–2010.

Consequently, it is believed that CRC incidence is rising in sub-Saharan Africa, although accurate figures remain largely elusive. The adoption of Western lifestyles, such as sedentary behaviour, obesity, smoking, and shifts from plant-based and fibre-rich diets to calorie-dense animal-based diets, may have significantly contributed to the escalating CRC rates [8]. According to Katsidzira *et al* [9], a population-based case-control study suggested that the traditional African diet appeared protective against CRC [9].

Risk factors associated with CRC in Zimbabwe were identified in a community-based case-control study, revealing associations with diabetes mellitus, prior urban domicile, previous schistosomiasis, and cancer in a first-degree relative [10].

Incidence rates in Sub-Saharan Africa generally increase with age in all registries from the African Cancer Registry Network, with rates often declining after age 75. Rates are generally higher in men than in women [3].

The oldest records of cancer in the African population in Mozambique were carried out by Prates and Torres [11], who conducted the cancer survey in the city of Lourenço Marques (now Maputo) from 1956 to 1961. They studied cases diagnosed in African Mozambican residents and non-residents in the city of Maputo, recording seven cases of colon cancer in men and four cases in women. For rectal cancer, there were three cases in men and two cases in women (Table 1) [11].

Since population-based CRC data remains limited in the region, any contribution to illuminate the extent of the CRC problem in Africa is valuable and significant.

With this in mind, we perform a study to investigate the evolution of CRC burden in Mozambique and its epidemiological characteristics, using data from the population-based oncological registries of Beira and Maputo, as well as the hospital-based registry of the Central Hospital in Maputo.

## Methods

**Figure 1.**
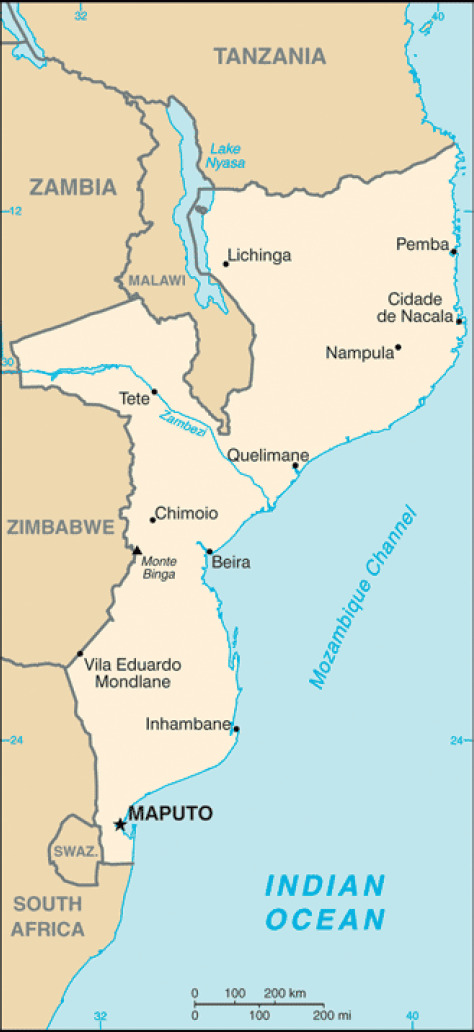
Maputo and Beira, Mozambique.

CRC data were compiled from the population-based cancer registries of Beira and Maputo, Mozambique (Figure 1). Consequently, data from the Maputo Population-Based Cancer Registries for the years 2015–2017 and data from the population-based cancer registry in Beira for the period 2018–2020 were initially examined separately and subsequently integrated into this study. A comparative analysis was conducted between data from the Lourenço Marques (now Maputo) Population-Based Cancer Registries for the years 1956–1961 and data from the Maputo population-based cancer registry for the years 2015–2017 [12].

In addition, this study incorporated supplementary information, including data from the Maputo pathology-based cancer registry and the Hospital Cancer Registry of Maputo Central Hospital (MCHCR). Information spanning from 1991 to 2008 was extracted from the Maputo pathology-based cancer registry [13], while data from 2015 to 2019 were obtained from the hospital-based MCHCR [14]. The analysis encompassed the period from 1956 to 2020, with a focus on investigating trends and epidemiological characteristics. The absence of personal identifiers rendered ethical approval unnecessary. The utilisation of the data was granted access permission by the relevant registries.

## Results

**Figure 2.**
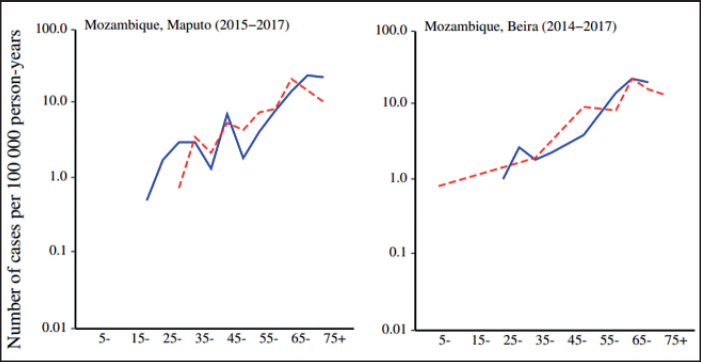
Age-specific incidence rates (per 100,000) of colon and rectum cancer, adapted from the cancer in Sub-Saharan Africa. Volume III can be found online at www.uicc.org and www.afcrn.org [3].

Our investigation revealed that the incidence rates of CRC in Maputo and Beira exhibit relatively low levels. Examining data from the cancer registry of Lourenço Marques (the former designation for Maputo) spanning 1956–1960, out of 600 registered cases, 87.3% underwent morphological verification. In the cancer registry of Lourenço Marques, CRC was slightly more frequent in females (with age-standardised rates of 1.94 per 100,000 for females and 1.93 per 100,000 for males) within the African population. Contrastingly, data originating from the Maputo cancer registry for the years 2015–2017 revealed an incidence rate of 3.17 per 100,000 inhabitants in males and 2.55 per 100,000 inhabitants in females (Table 2 and Figure 2). Meanwhile, the population-based cancer registry in Beira indicated an increase in standardised incidence rates for both genders from 2009 to 2017. Nevertheless, this pattern did not persist in the period spanning 2018–2020. The increase in incidence rates between 2009 and 2020 was particularly marked in the age groups 50 and above (Table 3).

Analysis of data sourced from the Maputo pathology-based cancer registry (covering 1991–2008) unveiled an annual percentage change (95% confidence interval) of 6.8% (with a range of 2.2–11.3) in males and 1.9% (ranging from - 3.7 to 7.5) in females. This indicates an increase in the number of diagnosed cases for both genders, with the most substantial growth rate observed among males.

**Figure 3.**
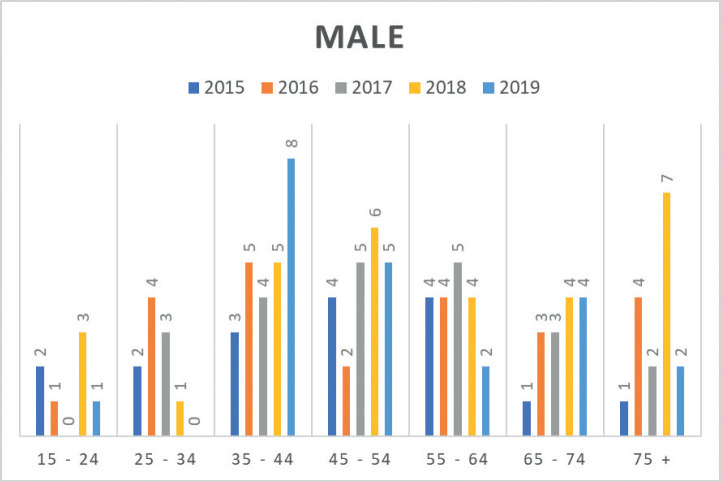
Number of CRC cases by age group in males (2015–2019) at MCHCR.

**Figure 4.**
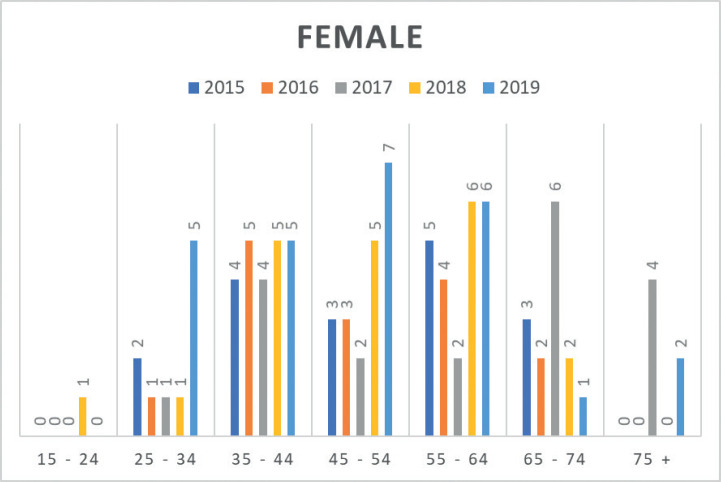
Number of CRC cases by age group in females (2015–2019) at MCHCR.

Moreover, insights from the hospital-based MCHCR highlighted a rise in diagnosed cases for both males and females between 2015 and 2019, with a slightly greater frequency among males. Over the period, colon cancer was slightly more common (51.7%) than rectal cancer in males (Table 4, Figures 3 and 4).

## Discussion

The findings align with several independent analyses showing a rising incidence of CRC in Africa [15].

Muluken Gizaw Turago, Assistant Professor at Addis Ababa University, presented on the topic of ‘Trends in the Incidence of Major Cancers in Sub-Saharan Africa’ at the 14th AORTIC International Conference on Cancer in Africa, held in Dakar from 3 November to 6 2023. The data, obtained from population-based cancer registries in Sub-Saharan Africa, specifically focused on CRC. He examined nine registries from Brazzaville, Gambia, Nairobi, Ibadan, Mauritius, Seychelles, Harare, Kampala and Easter Cape (South Africa). The findings revealed a rise in the age-standardised incidence rate of CRC across the mentioned regions.

To further explore this trend, we focused on CRC incidence in Mozambique using data from population-based cancer registers. Accurate evaluation of incidence trends based on cancer registry data requires consistent registration completeness over the study period [4]. While adherence to these principles cannot be guaranteed, audits of the population-based cancer registers in Beira and Maputo, as well as the hospital-based and pathology-based MCHCR, provide confidence in their relative completeness.

Recent data from the Maputo cancer registry (2015–2017) indicate standardised incidence rates of 3.17 for males and 2.55 for females. Incidence rates increase with advancing age. In Beira, age-standardised rates between 2009 and 2020 were 0.76 for males and 0.50 for females. The Beira population-based cancer registry indicated an increase in standardised incidence rates for both genders and incidence rates consistently grew in both genders within age groups over 50 years between 2009 and 2020 [3].

Trends in cancer incidence rates over the studied period in Mozambique may illustrate the impact of evolving lifestyles and the potential influence of the HIV/AIDS epidemic on CRC incidence, which increased during this period, as seen in other countries [9]. Similar CRC incidence trends have been observed in other sub-Saharan African countries [4–6, 7–8, 16–18].

The burden of non-communicable diseases (NCDs) in Mozambique has been on the rise, driven by changes in lifestyle factors such as smoking, alcohol consumption, diet, and physical activity. Of particular concern is the increasing prevalence of type 2 diabetes, overweight, and obesity, along with the persistently high burden of HIV/AIDS. Understanding the interplay between these lifestyle factors and NCDs is crucial for addressing the rising tide of CRC in Mozambique.

Between 2005 and 2014/2015, Mozambique witnessed a decline in the prevalence of daily smokers and smokeless tobacco users, indicating positive changes in tobacco consumption habits. However, there was a notable increase in the prevalence of overweight and obesity, especially in urban areas and among women. Concurrently, the prevalence of type 2 diabetes doubled, with men disproportionately affected. These trends underscore the importance of addressing modifiable risk factors for NCDs in Mozambique’s population [19–21].

Type 2 diabetes and obesity are established risk factors for CRC, with growing evidence linking these conditions to an increased risk of colorectal neoplasia. The rising prevalence of type 2 diabetes and obesity in Mozambique suggests a potential surge in CRC cases in the coming years. Additionally, the high burden of HIV/AIDS adds another layer of complexity, as individuals living with HIV may face an elevated risk of CRC due to immunosuppression and chronic inflammation [22].

Furthermore, Mozambique grapples with geographical disparities in health outcomes, including the prevalence of neglected tropical diseases (NTDs) such as schistosomiasis. Regions with high NTD burden coincide with areas of high CRC incidence, indicating potential synergistic effects between parasitic infections and CRC risk. Understanding the epidemiological overlap between NTDs and CRC could inform targeted screening and prevention strategies in endemic regions [23, 24].

However, incidence rates of CRC Maputo and Beira are low. Nevertheless, in Maputo, an increase in the annual percentage change in incidence rates (6% in men, 1.9% in women) was observed, although it did not reach statistical significance [14].

Cumulative incidence rates of colon and rectum cancer among males and females in sub-Saharan Africa, as reflected by registry populations in Maputo and Beira, are among the lowest in African cancer registries [3]. Despite this, we observed increasing incidence rates, particularly in age groups above 50 years and in both genders.

In a study conducted by Selemane *et al* [25] (2013–2016), which centred on CRC within the colorectal surgical service at Maputo Central Hospital, a higher prevalence was noted among females, with a median age of 54 years. The predominant histological type observed was adenocarcinoma, primarily manifesting in the rectum. Notably, most of the cases were diagnosed at advanced stages [25]. Gullickson *et al* [26] discovered similar findings while studying CRC survival across 13 population-based cancer registries in 11 African countries. The estimated 5-year relative survival rate of 48% in the cohort of CRC patients diagnosed between 2005 and 2015 was lower than survival rates reported in Western Europe in the 1980s and 1990s.

Data from MCHCR revealed that 36.1% of cases were diagnosed in individuals younger than 45 years, with no available family history of cancer. This lack of familial information prevents exploration of any potential association with genetic syndromes linked to CRC. Intriguingly, in a recent article, the author found that 5-year survival for patients aged 50–69 was 47.4%, compared to 38.8% in those under 50 and 40.9% in those aged 70 or older. The reason for lower survival in the younger age group remains unaddressed but suggests the possibility of more aggressive tumour types and delays in diagnosis [26]. This raises questions about whether biological factors might contribute to these differences [27–29].

The incidence of CRC is likely increasing, as suggested by our data, although the exact burden of the disease in Mozambique remains poorly understood, and access to definitive diagnosis and treatment has not been systematically quantified. Despite the challenges posed by high ambient temperatures, endemic parasitic infections, and feasibility concerns, the faecal immunochemical test (FIT) holds promise as a CRC screening tool for LMICs, including Mozambique. However, rigorous validation through prospective trials, consideration of concomitant parasitic testing, investment in healthcare infrastructure, cost-effectiveness analysis of screening, and public education are essential steps toward realising the potential of FIT-based CRC screening to reduce the burden of CRC in Mozambique [30].

The convergence of lifestyle factors, NCDs, infectious diseases, genetic syndromes and HIV/AIDS presents a multifaceted challenge to CRC prevention and control efforts in Mozambique. Addressing modifiable risk factors, improving access to screening and early detection services, and integrating CRC prevention into existing NCD and infectious disease programs are critical steps toward reducing the burden of this preventable cancer in Mozambique. Collaborative efforts involving policymakers, healthcare providers, researchers, and community stakeholders are essential for mitigating CRC risk and improving overall population health in Mozambique.

## Conclusion

Despite the relatively low cumulative incidence rates of colon and rectum cancer in Maputo and Beira, the observed increase in age-standardised incidence rates suggests a growing burden. Although more prevalent in ages over 45, a significant portion of diagnoses occur at younger ages. This phenomenon warrants dedicated and comprehensive investigation. The rising incidence rate emphasizes the need for early diagnosis programs, preventive measures for modifiable risk factors, secondary prevention strategies, investment in cancer infrastructure and policies, and workforce training tailored to addressing CRC.

## Conflicts of interest

The authors declared that they have no competing interests.

## Funding

The authors did not receive any external sources of funding

## Author contributions

**Conceptualisation**: Carlos Selemane, Mamudo Rafik Ismail and Lúcio Lara Santos. **Methodology**: Max Parkin and Lúcio Lara Santos. **Data curation writing and original draft preparation**: Carlos Selemane, Lúcio Lara Santos, Max Parkin. **Reviewing**: Carlos Selemane, Josefo Ferro, Cesaltina Lorenzoni, Carla Carrilho, Mamudo Rafik Ismail, Max Parkin and Lúcio Lara Santos. **Editing**: Lúcio Lara Santos.

## Figures and Tables

**Table 1.** Distribution of cases of malignant tumours by site, sex and age of Africans in Lourenco Marques (now Maputo) from 1 May 1956 to 30 April 1961.

Colon cancer cases							
Residents							
Age groups	35–39	40–44	45–49	50–54	55–59	60+	Total
Gender							
Male	1	1	0	0	1	1	4
Female	0	0	0	0	1	1	2
Non residents							
Male	0	0	1	1	1	0	3
Female	1	0	0	0	1	0	2
Rectal cancer cases							
Non residents							
Male	1	0	0	1	1	0	3
Female	0	0	2	0	0	0	2

**Table 2.** Incidence rate per 100,000 African population by age group and age-standardised incidence rates (1956–1961), Lourenço Marques (Now Maputo) and Maputo (2015–2017), Maputo, Mozambique.

Rate per 100,000 population						
		**Lourenco Marques (1956-1960)^a^**			**Maputo 2015-2017**	
*Age*	**Male**	**Female**	***BOTH***	**Male**	**Female**	***BOTH***
0–4	0.00	0.00	*0.00*	0.00	0.00	*0.00*
5–9	0.00	0.00	*0.00*	0.00	0.00	*0.00*
10–14	0.00	0.00	*0.00*	0.00	0.00	*0.00*
15–19	0.00	0.00	*0.00*	0.00	0.00	*0.00*
20–24	0.00	0.00	*0.00*	0.54	0.00	*0.26*
25–29	3.70	0.00	*1.90*	1.85	0.00	*0.94*
30–34	0.00	0.00	*0.00*	3.09	0.78	*1.94*
35–39	7.14	0.00	*3.40*	3.07	3.68	*3.39*
40–44	8.00	0.00	*3.85*	1.39	2.31	*1.89*
45–49	0.00	0.00	*0.00*	7.08	5.70	*6.32*
50–54	0.00	0.00	*0.00*	1.84	4.72	*3.39*
55–59	18.18	18.87	*18.52*	4.32	8.18	*6.31*
60+/60–64	0.00	10.81	*5.45*	8.64	8.69	*8.66*
65–69				14.55	21.86	*18.39*
70–74				23.32	0.00	*10.84*
75+				29.58	11.53	*18.64*
All ages (crude)	1.49	0.88	*1.21*	2.08	1.73	*1.90*
ASR(W)	**1.93**	**1.94**	***1.93***	**3.17**	**2.55**	***2.82***
ASR(W)-upper	3.92	4.70	*3.63*	4.33	3.51	*3.56*
ASR(W)-lower	−0.06	−0.82	*0.23*	2.01	1.58	*2.08*

a SOURCE: Cancer incidence in five continents Vol I

**Table 3.** Incidence rate per 100,000 population by age group and age-standardised incidence rates (2009–2020), Beira, Mozambique.

Age		2009–2013			2014–2017			2018–2020	
	**Male**	**Female**	***BOTH***	**Male**	**Female**	***BOTH***	**Male**	**Female**	***BOTH***
0–4	0.00	0.00	*0,00*	0.00	0.00	*0.00*	0.00	0.00	*0.00*
5–9	0.00	0.00	*0.00*	0.00	0.65	*0.33*	0.00	0.00	*0.00*
10–14	0.00	0.00	*0.00*	0.00	0.00	*0.00*	0.00	0.00	*0.00*
15–19	0.00	0.00	*0.00*	0.00	0.00	*0.00*	0.00	0.91	*0.45*
20–24	0.00	0.69	*0.35*	0.00	0.00	*0.00*	0.87	0.00	*0.44*
25–29	0.00	0.00	*0.00*	0.91	0.00	*0.46*	0.00	1.03	*0.52*
30–34	0.00	0.00	*0.00*	2.37	0.00	*1.25*	0.00	3.01	*1.45*
35–39	0.00	0.00	*0.00*	1.61	1.78	*1.69*	3.82	0.00	*2.01*
40–44	0.00	0.00	*0.00*	2.22	0.00	*1.12*	0.00	2.67	*1.33*
45–49	0.00	0.00	*0.00*	0.00	0.00	*0.00*	0.00	0.00	*0.00*
50–54	0.00	3.39	*1.59*	3.45	7.20	*5.28*	0.00	4.29	*2.19*
55–59	0.00	0.00	*0.00*	0.00	0.00	*0.00*	17.15	5.69	*11.41*
60–64	5.79	0.00	*3.06*	12.16	6.45	*9.39*	7.42	0.00	*3.76*
65–69	17.74	9.33	*13.64*	18.70	19.03	*18.86*	10.54	0.00	*5.20*
70–74	0.00	0.00	*0.00*	14.68	13.89	*14.28*	16.51	15.80	*16.15*
75+	0.00	0.00	*0.00*	0.00	9.24	*5.25*	0.00	0.00	*0.00*
All ages (crude)	0.24	0.24	*0.24*	0.97	0.79	*0.88*	0.95	0.84	*0.89*
ASR(W)	**0.76**	**0.50**	***0.64***	**1.96**	**1.82**	***1.91***	**1.93**	**1.26**	***1.60***
ASR(W)-upper	1.63	1.16	*1.19*	3.25	3.10	*2.82*	3.29	2.25	*2.44*
ASR(W)-lower	−0.10	−0.15	*0.09*	0.67	0.55	*1.00*	0.56	0.28	*0.76*

SOURCE: African Cancer Registry Network. Cancer in Sub-Saharan Africa Volume III

**Table 4.** Number of colon and rectal cancer cases diagnosed and registered (2015–2019), Maputo Central Hospital (MCHCR).

Year	Cancer	Male	Female	Total (CRC)	All cancers Diagnosed in MCHCR	CRC (%) in MCHCR
2015	Colon Rectum	9 10	12 6	21 16	1,653	2.2
2016	Colon Rectum	12 11	8 7	20 18	2,078	1.8
2017	Colon Rectum	13 9	5 14	18 23	1,788	2.2
2018	Colon Rectum	20 10	13 7	33 17	1,834	2.7
2019	Colon Rectum	12 10	12 16	24 26	1,988	2.5
